# Possible role of HPV/EBV coinfection in anoikis resistance and development in prostate cancer

**DOI:** 10.1186/s12885-021-08658-y

**Published:** 2021-08-17

**Authors:** Javid Sadri Nahand, Khadijeh Khanaliha, Hamed Mirzaei, Mohsen Moghoofei, Hossein Bannazadeh Baghi, Maryam Esghaei, Ali Reza Khatami, Maryam Fatemipour, Farah Bokharaei-Salim

**Affiliations:** 1 Department of Virology, School of Medicine, Iran University of Medical Sciences, Tehran, Iran; 2 Research Center of Pediatric Infectious Diseases, Institute of Immunology and Infectious Diseases, Iran University of Medical Sciences, Tehran, Iran; 3 Research Center for Biochemistry and Nutrition in Metabolic Diseases, Kashan University of Medical Sciences, Kashan, Iran; 4 Student Research Committee, Kashan University of Medical Sciences, Kashan, Iran; 5 Infectious Diseases Research Center, Kermanshah University of Medical Sciences, Kermanshah, Iran; 6 Infectious and Tropical Diseases Research Center, Tabriz University of Medical Sciences, Tabriz, Iran; 7 Immunology Research Center, Tabriz University of Medical Sciences, Tabriz, Iran

**Keywords:** Human papillomavirus, Co-infection, Epstein-Barr virus, Prostate cancer, Inflammation, Anoikis

## Abstract

**Background:**

This study aimed to evaluate the possible role of human papillomavirus (HPV) and Epstein–Barr virus (EBV) coinfection as an etiological factor for prostate cancer (PCa) development.

**Methods:**

This case-control study was conducted on 67 patients with PCa and 40 control subjects. The expression levels of cellular and viral factors involved in inflammation, tumor progression, and metastasis were quantified, using the enzyme-linked immunosorbent assay (ELISA) and quantitative real-time polymerase chain reaction (qRT-PCR) assay.

**Results:**

The EBV/HPV coinfection was reported in 14.9% of patients in the case group and 7.5% of the control subjects. The high-risk types of HPV, that is, HPV 16 and HPV 18, were responsible for 50 and 30% of HPV/EBV-coinfected PCa cases (*n* = 10), respectively. No significant relationship was observed between PCa and HPV/EBV coinfection (OR = 2.9, 95% CI: 0.18–45.2, *P* = 0.31). However, the highest percentage of HPV genome integration was found in the HPV/EBV-coinfected PCa group (8/10; 80%). Also, the mean expression levels of inflammatory factors (IL-17, IL-6, TNF-α, NF-κB, VEGF, ROS, and RNS), anti-apoptotic mediators (Bcl-2 and survivin), and anti-anoikis factors (Twist and N-cadherin) were significantly higher in the HPV/EBV-coinfected PCa group, compared to the non-coinfected PCa cases. Nevertheless, the tumor-suppressor proteins (p53 and pRb) and E-cadherin (inhibitor of anoikis resistance) showed significant downregulations in the HPV/EBV-coinfected PCa group, compared to the non-coinfected PCa cases.

**Conclusion:**

The HPV/EBV coinfection may be an etiological factor for PCa through modulation of cellular behaviors.

**Supplementary Information:**

The online version contains supplementary material available at 10.1186/s12885-021-08658-y.

## Background

Prostate cancer (PCa) is the second most common male cancer and the fifth leading cause of mortality worldwide [1]. However, there is limited information on the development, etiology, and pathogenesis of PCa. Various risk factors have been identified for PCa in men, including ethnicity, age above 50 years, infection, and acquired or inherited genetic mutations [2]. Chronic inflammation and infection may be associated with the development of cancer in various organs, including the thyroid, breast, stomach, liver, cervix, and colon. According to previous studies, inflammation is very common in the adult prostate [3–7]. Factors, such as dietary factors, hormonal changes, cell damage, and infection (especially sexually transmitted infections), are some of the etiological factors that contribute to the initiation of prostatic inflammation. Besides, epithelial cellular injury due to chronic inflammation may lead to the loss of tolerance in normal prostate-associated antigens and trigger a sustained autoimmune reaction [3, 8].

Of approximately 1400 human pathogens, 220 are related to viral agents. A limited number of these viral agents, which are associated with cancer in various ways, are called oncogenic viruses. A recent theory suggests that virus-induced mechanisms can alter the biology and aggression of many cancers. Also, tumor behaviors alter under the influence of virus-induced mechanisms in cancer and stromal cells in the tumor environment. Some of these changes include the inhibition of cancer cell apoptosis pathways, changes in tumor metabolism, inhibition of anti-tumor immune system, provoked inflammation of the tumor environment, onset of angiogenesis, induction of tumor cell proliferation, increased tumor invasion, and increased metastasis [9]. Following the detection of several pathogens, including Epstein-Barr virus (EBV) and human herpes simplex virus type 2 (HSV2) [2] s and herpesviruses, including cytomegalovirus (CMV) and human papillomavirus (HPV) [10], in the prostate tissue, researchers have suggested that viral infections may influence the PCa development pathways [3, 10]. However, there is no evidence to prove the direct correlation of infection frequency and inflammatory response with PCa carcinogenesis [3, 10]. The EBV and HPV are well-established oncoviruses, which can initiate various human carcinomas [11–13]. In high-risk HPV types, E5, E6, and E7 proteins interfere with tumor-suppressor proteins in cells [14]. Besides, HPV E6 and E7 oncoproteins can modify the tumor milieu by regulating certain pro-inflammatory chemokines and cytokines, which can in turn affect the host immune response [15]. On the other hand, EBV, encoding EBV nuclear antigen 1 (EBNA1) and latent membrane protein-1 (LMP1), plays the most important role in EBV-related carcinogenesis by preventing apoptosis and stimulating cell survival, motility, proliferation, and angiogenesis [16, 17].

Approximately 12% of all cancers are caused by viral infections worldwide, with HPV and EBV accounting for 38% of virus-associated cancers [18]. Both EBV and HPV can be transmitted through sexual contact [19]. Consequently, the likelihood of EBV and HPV co-existence among cancer patients is increasing [18]. Numerous studies have reported EBV-HPV coinfection in some carcinomas [20–25]. Generally, the presence of both HPV and EBV sequences in healthy, malignant, and benign prostate samples [26–29] is of great importance, as many experimental findings have shown that HPV and EBV may play a role in the development of PCa [30].

It has been shown that coinfections increase the host’s susceptibility to cancers by affecting the immune responses. They mostly act as cofactors in cancer development and affect cancer behaviors in response to treatment [31]. However, the potential role of EBV/HR-HPV coinfection in the development of PCa is unclear. Therefore, the current study aimed to evaluate the association of EBV/HPV coinfection with PCa development, mediated by deregulation of cellular events linked to inflammation (reactive oxygen species [ROS], reactive nitrogen species [RNS], transforming growth factor-β [TGF-β], vascular endothelial growth factor [VEGF], interleukin-17 [IL-17], IL-11, IL-8, IL-6, IL-1, tumor necrosis factor-α [TNF-α], and NF-kB) and tumor progression (N-cadherin, Rb, P53, survivin, B-cell lymphoma-2 (Bcl-2), CD44, Twist, Slug, PTPN13, and E-cadherin). Besides, the expression levels of viral genes (E7, E6, E2, LMP-1, LMP-2, EBV-encoded RNA 1 [EBER1], and EBER2) and the association of these viral genes and cellular factors with PCa development were investigated in this study.

## Methods

### The collection of samples

The present multicenter case-control research was conducted during December 2018 to April 2020. First, 67 prostate tumor samples (*n* = 67) were obtained considering the inclusion and exclusion criteria during the study period in Tehran, Iran. Moreover, healthy tissues (*n* = 40) dissected from the peripheral area of adenoma removed by surgical procedure were collected as control (in terms of age). At least 24 h prior to surgery, serum samples were prepared from blood by venipuncture and stored at − 80 °C. Additionally, the tumor stage was detected based on the TNM system in accordance with the consultation of a team of experts in cancer including a cancer surgeon, a radiologist and an oncologist. The ethical considerations were in accordance with the Helsinki Declaration, and both verbal and written informed consents were achieved from all research units. Snap-frozen tissue samples in liquid nitrogen were stored at − 80 °C. The clinical profiles of patients included tumor stage, tumor type and age (Table 1).

**Table 1:** Comparison of the characteristics of research units between prostate cancer and control groups

Characteristics		Prostate Cancer (67)	Control (40)	P	OR (95% CI)
Age (Year)		52.7 ± 12.2	55.6 ± 9.9	0.19	0.95 (0.9–1.02)
HPV positive samples	Presence	31.3% (*n* = 21)	15% (n = 6)	0.060	1.84 (0.08–0.6)
EBV positive samples	Presence	49.3% (*n* = 33)	40% (*n* = 16)	0.353	1.06 (0.8–1.39)
Mono HPV-infection		16.4% (11)	7.5% (n = 3)	0.289	1.06 (0.26–6.5)
Co-infection (EBV&HPV)		14.9% (n = 10)	7.5% (n = 3)	0.314	2.9 (0.18–45.2)
Mono EBV-infection		34.3% (*n* = 23)	42.5% (*n* = 17)	0.48	0.58 (0.17–2.6)
Non-EBV and Non-HPV samples		34.3% (n = 23)	42.5% (n = 17)	0.48	NA
HPV Genotype	6	4.8% (1/21)	0	0.248	1.23 (0.51–4.42)
	11	0	16.7% (1/6)		
	16	47.6% (10/21)	50% (3/6)		
	18	33.3% (7/21)	33.33% (2/6)		
	33	3 (15.8%)	0		
EBV Genotype	1	9.09% (3/33)	0	0.105	0.55 (0.077–3.71)
	2	90.9% (30/33)	100% (20/20)		
Type of Cancer	Acinar adenocarcinoma	40 (59.7%)	–	NA	NA
	Ductal Adenocarcinoma	21 (31.34%)	–	NA	NA
	Squamous cell cancer	6 (8.95%)	–	NA	NA
Stage of Cancer	I	4 (5.9%)	–	NA	NA
	IIA	9 (13.4%)	–	NA	NA
	IIB	9 (13.4%)	–	NA	NA
	IIC	5 (7.4%)	–	NA	NA
	IIIA	2 (2.98%)	–	NA	NA
	IIIB	10 (14.9%)	–	NA	NA
	IIIC	6 (8.9%)	–	NA	NA
	IVA	12 (17.9%)	–	NA	NA
	IVB	10 (14.9%)	–	NA	NA

*NA* Not applicable

The study was approved by ethical committee of the Iran University of Medical Sciences (IUMS), Tehran, Iran, under the Ethics code of IR.IUMS.REC.1398.642. As well as, the current study was supported by grant no, 15671 from the research deputy of Iran University of Medical Sciences (IUMS).

### Detection of HPV and EBV by PCR

Total DNA was extracted from the tissue samples by the QIAamp DNA Mini Kit based on the kit protocol from the frozen samples of PCa. The quality of extracted DNA was analyzed using a 268-bp fragment amplification of the b-globin gene with the aid of HotStarTaq DNA polymerase (Qiagen, Dusseldorf, Germany) in the presence of G074 (5′-CAACTTCATCCACGTTCACC- 3′) and G073 (5′-GAAGAGCCAAGGACAGGTAC- 3′) primers. The cycling program was 95 ֯C for 9 min, and then 35 cycles at 95C for 30 s, 55 ֯C for 30 s, 72 ֯C for 1 min, and a final extension at 72 ֯C for 10 min [32].

The DNA extracted from 67 and 40 fresh frozen prostate cancer and control samples, respectively were analyzed by PCR for identification of HPV-L1 gene (MY11: 5′-GCMCAGGGWCATAAYAATGG-3′ and MY09: 5′-CGTCCMARRGGAWACTGATC-3′) and for detection of HPV E6/E7 gene (pU-H: 5′- GAGCTGTCGCTTAATTGCTC-3′ and pU-R: 5′-TGCTAATTCGGTGCTACCTG-3′) [33].

For EBV detection nested-PCR method was used by primers that used in Breda et al. study [34], including 5′-GCGGGTG-GAGGGAAAGG-3′ and 5′-GTCAGCCAAGGGACGCG-3′ for the first PCR round as well as 5′-GCCACCTGGCAGCCCTAAAG-3′ and 5′-AGGCTGCCCACCCTGAGGAT-3′ for the second PCR round [34]. Ten nM of each primer (Metabion, Germany), 2X Taq Master Mix, 1 μg of template DNA, and water at a final volume of 25 μl. The amplification of the samples was performed by 45 PCR cycles with the thermal program as follows; (1) The first PCR reaction: the initial denaturation for 5 min at 94 °C; for 30s at 94 °C; for 30s at 57 °C and for 1 min at 72 °C, the final extension at 72 °C for 7 min, (2) the second PCR reaction: the initial denaturation for 5 min at 94 °C, for 30 s at 94 °C, for 30 s at 50–57 °C and for 1 min at 72 °C, and the final extension at 72 °C for 7 min.

### EBV and HPV genotyping

In EBV positive samples, the EBV typing was performed by the EBNA2 primers [35]. The size of PCR products expected for EBV strains 1 and 2 is respectively 300 and 250 bp [35].

The samples positive for HPV were genotyped using INNO-LiPA HPV Genotyping v2 test (Innogenetics, Ghent, Belgium) based on the kit protocol.

### Quantitative real-time PCR

The expression of cellular gene (SLUG), HPV genes (E7, E6, and E2) and EBV genes (EBER 1, EBER-2, LMP-1 and LMP-2) was quantified by real-time PCR in the HPV-EBV-positive and HPV-positive PCa samples, respectively. To this end, the extraction of total RNA (1 μg) was performed by QuantiNova Reverse Transcription Kit (QIAGEN, Germany), followed by constructing the cDNA in a thermal cycler as 27 °C for 10 min, 38 °C for 15 min, 44 °C for 40 min, and 72 °C for 15 min. (The reaction condition was set as followed: 45 °C for 2 min in the DNA elimination reaction, 25 °C for 3 min in the annealing phase, 45 °C for 10 min in the reverse transcription phase and 5 min 85 °C in the inactivation of reaction).

#### EBV genes

##### LMP-1

The expression level of LMP-1 in EBV-infected PCa tissue was measured by qRT-PCR technique by the probes and primers according to Kubota et al. [36].

##### LMP-2A

Quantitative real-time PCR was carried out in EBV-positive tissues for LMP-2A by QuantiNova SYBR Green PCR kit (Qiagen, Hamburg, Germany) which has been described in Busson et al. [37].

##### Eber

The EBER1 and EBER2 levels were measured in EBV-positive PCa tissue using qRT-PCR according to Shannon-Lowe et al. [38].

#### HPV genes

QuantiNova Reverse Transcription® Kit, one step RT-PCR® kits (QIAGEN, Hilden, Germany) and Quantitative SYBR green TaqMan Universal PCR Master Mix® (QIAGEN, Germany) were respectively applied to recognize the viral E7, E6 and E2 genes. The serial dilutions of E7, E6 and E2 genes cloned in PUC57 vector (GenScript, Jiangsu, China) were utilized for viral genes, which contained equivalent volumes of these genes from 72 to 865 million copies/reaction, as control.

The viral genes of E2 (forward primer: 5′-CTACGAATTCATGGAGACTCTTTGCCAACG-3′ and reverse primer: 5′-GATAGAATTCTCATATAGACATAAATCCAG-3′), E6 (forward primer: 5′-GCAATGTTTCAGGACCCACA-3′ and reverse primer: 5′-ACAGCATATGGATTCCCATCTC-3′) and E7 (forward primer: 5′-AAGTGTGACTCTACGCTTCGGTT-3′, reverse primer: 5′-GCCCATTAACAGGTCTTCCAAA-3′ and Probe of FAM-TGCGTACAAAGCACACACGTAGACATTCGTA-BHQ), were respectively detcted using one step RT-PCR® kits (QIAGEN, Hilden, Germany), QuantiNova Reverse Transcription® Kit and Quantitative SYBR green TaqMan Universal PCR Master Mix® (QIAGEN, Germany) [39]. The serial dilutions of E7, E6 and E2 genes cloned in PUC57 vector (GenScript, Jiangsu, China) were utilized for viral genes, which contained equivalent volumes of these genes from 72 to 865 million copies/reaction, as control.

#### Cellular gene (SLUG)

The SLUG gene was amplified by the qRT-PCR in the presence of specific primers. The real time PCR device (Rotor-Gene® Q; Qiagen, Hilden, Germany) was utilized exploiting the Power SYBR Green PCR Master Mix (TaKaRa Bio; Kusatsu; Japan). The normalization of the relative expression level for the gene was carried out by a GAPDH as housekeeping gene. The sequences of F and R primers to amplify the SLUG gene were as follows: the forward primer sequence was as: 5′-GCCTCCAAAAAGCCAAACTACA-3′, the reverse primer sequence was as: 5′-GAGGATCTCTGGTTGTGGTATGACA-3′ [40].

In addition, the expression level was measured by the equation of 2^(−ΔΔCt)^ exploiting the online data analysis tool of QIAGEN (Gene Globe; http://www.qiagen.com/us/ shop/genes-and-pathways/data-analysis-center-overview-page/). All reactions were repeated three times, and the internal control was considered to be GAPDH for the normalization of gene expression level (E7, E6, and E2) for various specimens.

### Physical status of HPV DNA

The qPCR technique was used to compute the E2/E6 ratio for the characterization of the HPV-DNA physical status (episomal or integral status) in the specimens infected with the HPV. The E2/E6 = 0 was defined as a fully integrated status and the E2/E6 ≥ 1 as an episomal status, and the E2/E6 = 0–1 as a mixed status [41].

### Enzyme linked immunosorbent assay (ELISA)

#### Estimation of serum cytokines and survivin levels

The taken whole blood was poured into the SST™ serum separation tubes. Each sample had five complete inversions. The blood was left at room temperature for 15–30 min in order to clot. No longer than 2 h after collecting, the separator gel tubes had centrifugation, followed by discarding the clot for 15 min at 1500×g. The serum samples were distributed aseptically at 50-μl volumes and kept − 80 °C for subsequent testing.

The serum levels of IL-17, IL-11, IL-8, IL-6 and IL-1 were measured by the related ELISA kit (Abcam, Cambridge, MA, USA) based on the suggested protocol. Moreover, the serum levels of VEGF, TGF-β and TNF-α were measured by Quantikine Assay Kit (R&D Systems), Human TGF-β Quantikine ELISA® Kit (Minneapolis) and Human TNF-α PicoKine ELISA Kit (Boster), respectively, based on the kit protocols.

The survivin (anti-apoptotic mediator) expression level was measured by Survivin Human SimpleStep ELISA® Kit (Abcam) based on the manufacturers’ protocol.

#### Measurement of Bcl-2 and anoikis-related factors in tissue samples

In brief, all prostae tissue samples were grounded individually in the liquid nitrogen, and then lysed by BioPlex lysis buffer (Bio-Rad, Hercules, CA) inside the microcentrifuge tubes. The tissue lysate was homogenized in a Dounce homogenizer, followed by centrifugation with 13,000 rpm for 10 min at a temperature of 4 °C to obtain a clear supernatant containing the lysate of prostate tissue.

The Bcl-2 level was quantified in all tissue lysate samples by the Human Bcl-2 ELISA Kit (Abcam) based on the related kit protocol.

The expressin level of anoikis-related proteins including PTPN13, TWIST, N-cadherin and E-cadherin in prostate tissue lysates were measured using Human Tyrosine-Protein Phosphatase Non-Receptor Type 13 (PTPN13) ELISA Kit (MyBioSource, USA), TWIST ELISA Kit (Aviva Systems Biology, CA, USA) and Human E-Cadherin, N-Cadherin ELISA Kit (Abcam, Cambridge, MA, USA) respectively, based on the related kit protocols.

#### Quantification of reactive oxygen/nitrogen species, p53, retinoblastoma and NF-kB in prostate tissue lysates

The level of ROS and RNS were determined in tissue lysate samples by the OxiSelect™ Intracellular ROS/RNS Assay Kit (Cell Biolabs, Inc., San Diego, CA), based on the kit protocol.

Quantification of NF-kB, Rb and p53 was performed in the tissue lysates by NF-kB p65 Transcription Factor Assay® Kit, Human Retinoblastoma ELISA® Kit (Sigma-Aldrich, Saint Louis, USA) and Abcam’s p53 Simple Step ELISA® Kit (Cambridge, USA), respectively, based on the kit protocols.

### Statistical methods

Statistical analysis was performed by GraphPad Prism 6 and STATA versions 11.2 software. Kolmogorov–Smirnov test was used to assesse the normality of data distribution. Data were reported as mean ± standard deviation (SD) of parametric variables, and the median and the interquartile range for nonparametric data. In the comparison of central tendency parameters, including the mean for the normal variables and the median for the non-normal variables, two-independent samples t-test or Mann-Whitney non-parametric tests were applied between two groups, as well as one-way ANOVA or kruskal-wallis tests were applied between more than two groups. Fisher’s exact test or Chi-square was utilized to evaluate the associations between the categorical data. Spearman’s rank correlation was measured for obtaining the extent of coexpression between the cellular factors and the viral genes. The statistically sinificance level was considered to be *P* < 0.05, adjusted by Bonferroni’s correction test for ANCOVA analysis of data. Benjamini-Hochberg method was applied to correct the false discovery rate for multiple comparisons. Heat maps were plotted by one matrix CIMminer using Ward cluster algorithm and Euclidean distance approachs.

## Result

### The profiles of research units

The demographic profiles of the study participants (*n* = 107) are shown in Table 1. Totally, 67 PCa cases and 40 control were collected between August 2018 and March 2020. The cases and controls were homogeneous for the age (*P* = 0.19). The observed types of PCa tissues included acinar adenocarcinoma (40/64, 59.7%), ductal adenocarcinoma (21/64, 31.3%) and squamous cell cancer, SCC (6/64, 8.9%). In the current study, the lowest and highest cancer stages were respectively IIIA (2.98%) and IVA (17.9%). EBV-DNA was found among 49.3% of PCa samples, HPV DNA in 31.3%, while in control group in 40 and 15%, respectively. Among 67 PCa tissue specimens, HPV mono-infection was detected in 11 (16.4%), EBV mono-infection was detected in 23 (34.3%), and HPV/EBV co-infection was detected in 10 (14.9%). As well as, among 40 control tissue specimens, HPV mono-infection was detected in 3 (7.5%), EBV mono-infection was detected in 17 (42.5%), and HPV/EBV co-infection was detected in 3 (7.5%). There were not significant association between the presence of EBV, HPV and HPV/EBV co-infection with PCa (OR = 1.06, 95%CI = 0.8–1.39, *P* = 0.35, OR = 1.84, 95%CI = 0.08–0.6, *P* = 0.06, and OR = 2.9, 95%CI = 0.18–45.2, *P* = 0.34, respectively). HPV 16 and EBV 2 were the highest genotypes isolated in both case group (47.6 and 90.9%, respectively) and control group (50 and100%, respectively). The most frequent HPV genotypes in HPV/EBV co-infected positive groups were HPV-16 (6/11, 54.54%), followed by HPV-18 (3/11, 27.24%), HPV-33 (1/11, 9.09%), and HPV-6 (1/11, 9.09%) (Table 1). Also, there was no significant association between all HPV and EBV genotypes and the PCa development (*P* = 0.24 and 0.1, respecitvely). No statistically association was reportd between EBV/HPV co-infection and histopathological types of tumor (*P* = 0.268, and 0.353). No significant difference was seen in the frequency distributions of PCa stages between the EBV-positive PCa and the EBV-negative PCa, as well as between the HPV-positive PCa and the HPV-negative PCa (*P* = 0.17 and 0.54).

### Physical status of the HPV genome

The HR-HPV genome integration in the host chromosome importantly deregulates the expression of E6 and E7 oncogenes, thereby resulting in cell transformation [42]. It should be noted that the association of integration with contributing to prostate malignancy has not been the reported. A fully integrated type was seen in 45% of PCa group and 20% of control group. In 10% of PCa and 20% control groups, HPV genome was present in a purely episomal form. Also, mixed forms (episomal and integrated genomes) were found in 50 and 80% of the PCa and control specimens, respectively (Table 2). Furthermore, the frequency of the fully HPV genome integrated type was significantly higher in the PCa group co-infected with the HPV/EBV when comparing with the mono-HPV infected PCa group (*P*: 0.0009 and 0.002, respectively). It is noteworthy that all integrated form of HPV DNA was found in the HPV/EBV co-infection samples. The HPV 16 DNA integrated type was observed in 5 of 20 (25%) HPV-positive PCa, in 5 of 10 (50%) HPV/EBV-co-infected positive PCa and in 1 of 1(100%) HPV/EBV co-infected positive control groups. The HPV-18 DNA was integrated in 3 of 21 (14.2%) HPV-positive PCa, and in 3 of 10 (30%) HPV/EBV-co-infected positive PCa groups (Table 2).

**Table 2:** The HPV genome physical state in the studied groups

Status	PCa group	Controls group	Mono HPV infected PCa group	Co-infected PCa group	Mono HPV infected (%) Control group	Co-infected Control group
Fully integrated state (E2/E6 = 0)	9/20 (45%)	1/5 (20%)	1/11 (9.09%)	8/10 (80%)	0	1/3 (33.33%)
	P: 0.86		P: 0.0009		P: NA	
Episomal state (E2/E6 = 1)	2/20 (10%)	1/5 (20%)	2/11 (18.18%)	0	0	1/3 (33.33%)
	P: 0.62		P: NA		P: NA	
Mixed state (E2/E6 = > 0 to < 1)	10/20 (50%)	4/5 (80%)	8/11 (72.72%)	2/10 (20%)	3/3 (100%)	1/3 (33.33%)
	P:0.74		P: 0.003		P: 0.002	

*NA* Not applicable

### The comparison of expression pattern of viral genes between study groups

**Fig. 1:**
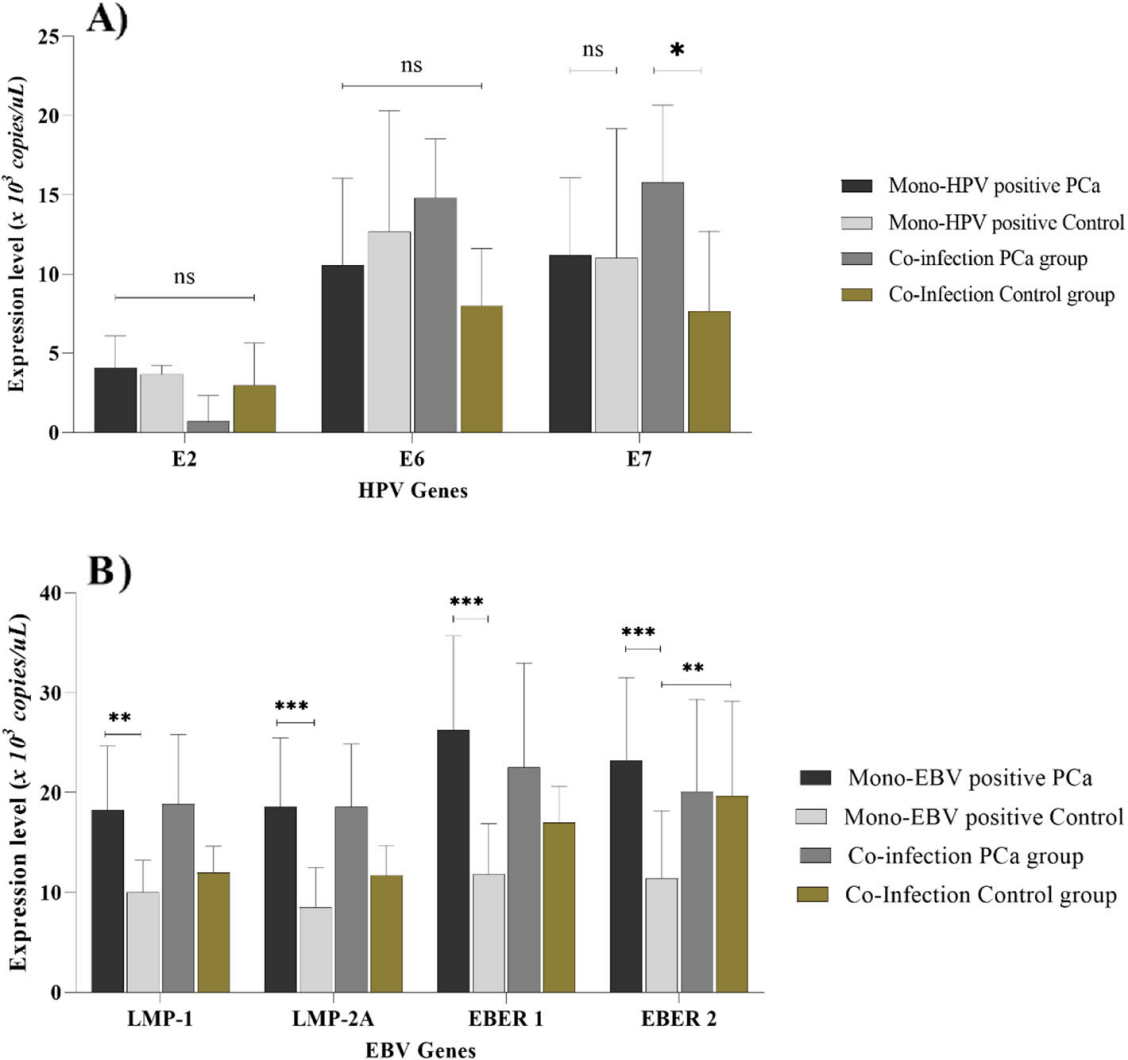
Differences in the expression level of viral genes in the two mono-infection and co-infection groups. NS: not significant at the level of 0.05. (* *P* ≤ 0.05, ** *P* ≤ 0.01, *** *P* ≤ 0.001)

Table 3 shows the expression level calculated for the EBV genes (EBER-1, EBER-2, LMP-1 and LMP-2) and HPV genes (E2, E6 and E7) in both stages and types of PCa. The maximum expression level of EBV genes examined was EBER 2 in stage IIA samples (mean ± SD:22.4 ± 9.7) as well as the lowest expression level of EBV genes was that of LMP 2 in stage IIIC samples (mean ± SD: 7.1 ± 8.3). The lowest and highest expression of LMP-1 gene were observed in stage IIIB (mean ± SD: 7.5 ± 10.2) and IVB (mean ± SD: 15 ± 11.9) samples, respectively. The expression of both LMP-2 and EBER-1 had the maximum mean levels in stage I specimens, and EBER 2, has the lowest expression in stage IIIB samples. Stratification of the specimens in terms of the types of cancer demonstrats the EBER 1 level in SCC (mean ± SD: 18.6 ± 7.5) and EBER 2 in ductal adenocarcinoma (mean ± SD: 8.7 ± 9.5) samples were respectively the maximum and the minimum. The LMP-1 and LMP-2 gens had the highest expression level in acinar adenocarcinoma and SCC samples, respectively (Table 3). Figure 1B shows that a significant difference was in the EBV gene expression between mono EBV-positive PCa samples, whereas, except for EBER-2 gene, no significant difference was found between the mono EBV-positive PCa and HPV/EBV-coinfection positive PCa samples.

**Table 3:** Comparison of the expression level of HPV and EBV genes between types and stages of Prostate Cancer

Cancer characteristic		E2	E6	E7	LMP-1	LMP-2	EBER 1	EBER 2
Types of Cancer	Acinar adenocarcinoma	2.5 ± 2.5	13.1 ± 6.2	12.89 ± 6.3	14.2 ± 7.4	12.3 ± 11.3	17.8 ± 15.3	16.2 ± 10.2
	Ductal adenocarcinoma	2.4 ± 2.8	12.91 ± 5.9	13.87 ± 4.63	13.07 ± 11.4	9.7 ± 10.8	8.6 ± 10.1	8.7 ± 9.5
	Squamous cell cancer	0	17.33 ± 4.7	19.17 ± 4.3	11.8 ± 2.7	13.5 ± 11.1	18.6 ± 7.5	17.2 ± 8.1
	P	Ns	ns	ns	Ns	ns	ns	ns
Stages of Cancer	I	0	0	0	14.5 ± 9.2	17.5 ± 12.1	20.1 ± 11.5	19.8 ± 10.8
	IIA	0	13 ± 2.8	14 ± 4.2	15 ± 9.50	17.4 ± 9.6	20.3 ± 8.2	22.4 ± 9.7
	IIB	3 ± 1.85	6.6 ± 2.7	7.3 ± 1.5	10.8 ± 5.33	7.6 ± 12.6	9.8 ± 16.3	8.21 ± 4.04
	IIC	0	0	0	12.8 ± 6.9	12.8 ± 11.9	17.3 ± 7.2	15 ± 5.11
	IIIA	0	0	0	8 ± 6.31	11 ± 3.2	9.8 ± 11.2	12.3 ± 10.4
	IIIB	4.5 ± 1.99	13.8 ± 4.9	11.6 ± 4.8	7.5 ± 10.2	7.9 ± 7.3	16.8 ± 5.9	11.3 ± 4.8
	IIIC	3 ± 0.54	14.5 ± 7.5	17 ± 1.8	9.8 ± 9.01	7.1 ± 8.3	8.9 ± 9.02	10.8 ± 3.3
	IVA	5.4 ± 2.8	12.7 ± 4.3	13.4 ± 3.9	12 ± 11.7	15.1 ± 11.8	11.5 ± 7.2	12.6 ± 4.52
	IVB	0	15.5 ± 2.7	17.2 ± 2.5	15 ± 11.9	16.5 ± 11.2	17.2 ± 7.7	13.7 ± 6.2
	P	Ns	ns	ns	ns	ns	ns	ns

Geometric Mean ± Standard Deviation, NA: Not available, ns: not significant

The lowest and highest levels were found for both E6 and E7 genes in stage IIB (mean ± SD: 6.6 ± 2.7 and 7.3 ± 1.5, respectively) and IVB (mean ± SD: 15.5 ± 2.7 and 17.2 ± 2.5, respectively) samples, respectively (Table 3). The minimum HPV gene expression level was related to E2 that was observed in the stage IIIC (mean ± SD: 3 ± 0.54) samples. Concerning the cancer type, the E7 gene expression level were the highest in the SCC (mean ± SD: 19.17 ± 4.3), as well as, the highest E6 gene level was observed in the SCC. According to Fig. 1, the expression level of HPV genes between the mono HPV-positive PCa cases and the mono HPV-positive controls as well as between the mono HPV positive PCa cases and the HPV/EBV coinfected PCa cases was not significantly different. Nonetheless, the expression level of E7 gene was significantly higher in the HPV/EBV coinfected PCa group when comparing with the HPV/EBV coinfected controls.

### Comparison of expression pattern of inflammatory-related factors among study groups

We examined the expression levels of different inflammatory mediators between the PCa samples with the control samples and between EBV/HPV-positive samples with EBV/HPV-negative samples. The result is shown in Tables 4, 5, and 6. According to the results obtained, the level of inflammation-related factors including NF-κB, TNF-α, IL-17, IL-11, IL-8, IL-7, IL-6, IL-1, VEGF, TGF-β, ROS, and RNS in the PCa group higher than in the control group. For detection of whether HPV and/or EBV infection or/and HPV/EBV-coinfection are implicating in regulating inflammation in PCa, we analyzed the expression level of these factors among EBV infected, HPV infected, and HPV/EBV coinfected samples. All inflammatory factors mentioned above was higher significantly in the groups positive for HPV compared to the groups negative for HPV (Table 5). Furthermore, as shown in the Table 5, the maximum levels of expression were seen for the ROS (*P* < 0.0001, fold change: 7.5), RNS (P < 0.0001, fold change: 2.6) and IL-6 (P < 0.0001, fold change: 2.51) in the PCa group positive for HPV compared with the PCa group negative for HPV. The correlation results in Table 7 showed significantly positive correlation between the expression level of the viral proteins (E2, E6 and E7) and the inflammatory agents (*P* < 0.001). The strongest positive correlation was between ROS and E6/E7 (r = 0.763, *P* = 0.0001), and then between Survivin and E6/E7 (r = 0.75, r = 0.76, respectively, *p* value = 0.0001 for both).

**Table 4:** Comparison of cellular factors levels between the EBV positive and negative samples

Cellular Factors	PCa group (a) vs control group (b)	EBV-positive samples (c) vs EBV-negative samples (d)	EBV-positive PCa (e) vs EBV-negative PCa (f)	EBV-positive control (g) vs EBV-negative control (h)	P^*^	P^+^	P^$^	P^
NF-κB	a: 19.4 ± 7.4 b: 9.5 ± 7	c: 18 ± 7.8 d: 12.7 ± 9	e: 22.4 ± 4.3 f: 15.4 ± 8.8	g: 10.9 ± 7 h: 7.1 ± 6.6	< 0.0001	0.006	0.004 1.45	ns
TNF-α	a: 17 ± 7.4 b: 11.7 ± 6.7	c: 18 ± 5.5 d: 11.03 ± 8.1	e: 19.7 ± 5.7 f: 13.4 ± 7.9	g: 15.2 ± 4.1 h: 6 ± 6.4	0.002	< 0.0001	0.001 1.47	0.001
IL-1	a: 13 ± 5.7 b: 7.8 ± 5.3	c: 13.1 ± 5.3 d: 8.4 ± 6.1	e: 15 ± 4.6 f: 10.4 ± 6	g: 10 ± 4.8 h: 4.2 ± 4	0.0002	0.001	0.01 1.44	0.05
IL-6	a: 14.4 ± 6 b: 8.9 ± 6.6	c: 14.8 ± 5.8 d: 9 ± 6.6	e: 16.4 ± 5.5 f: 11.8 ± 5.9	g: 12.3 ± 5.5 h: 3.4 ± 3.9	0.0004	0.0003	0.02 1.3	0.004
IL-8	a: 12.62 ± 6.2 b: 5.68 ± 5.1	c: 10.4 ± 6.6 d: 9.7 ± 6.8	e: 12.7 ± 6.3 f: 12.4 ± 6.01	g: 6.7 ± 5.5 h: 4.1 ± 3.7	< 0.0001	ns	ns	ns
IL-11	a: 15.6 ± 8.3 b: 7.1 ± 6.2	c: 13.4 ± 8.6 d: 11.3 ± 8.5	e: 16.5 ± 8.3 f: 14.3 ± 8.2	g: 8.3 ± 6.4 h: 5.5 ± 5.2	< 0.0001	ns	ns	0.049
IL-17	a: 9.8 ± 3.9 b: 4.3 ± 3.8	c: 8.2 ± 4.8 d: 7.4 ± 4.6	e: 10.2 ± 4.1 f: 9.4 ± 3.7	g: 4.9 ± 4.01 h: 3.3 ± 3.6	< 0.0001	ns	ns	ns
VEGF	a: 38.64 ± 10.8 b: 13.7 ± 6.9	c: 33.5 ± 14 d: 27.9 ± 15.07	e: 40.8 ± 8.5 f: 32.8 ± 14.3	g: 23 ± 12.4 h: 14.05 ± 6.08	< 0.0001	0.03	0.01 1.2	0.02
TGF-β	a: 15.2 ± 7.6 b: 7.5 ± 5.1	c: 13.5 ± 7.9 d: 10.9 ± 7.3	e: 16.4 ± 7.8 f: 13.6 ± 7.1	g: 8.7 ± 5.3 h: 5.4 ± 4.2	< 0.0001	ns	ns	0.039
Rb	a: 9.1 ± 5.7 b: 14.06 ± 6.4	c: 10.7 ± 6.2 d: 11.1 ± 6.8	e: 9.4 ± 6.1 f: 8.8 ± 5.4	g: 13 ± 5.8 h: 15.8 ± 7.3	0.0006	ns	ns	ns
P53	a: 10.4 ± 6.7 b: 13.9 ± 6.6	c: 10.8 ± 6.3 d: 13 ± 7.4	e: 10.1 ± 6.01 f: 10.9 ± 6.8	g: 11.9 ± 5.6 h: 17.3 ± 6.9	0.04	ns	ns	0.02
ROS	a: 10.2 ± 9.7 b: 6.09 ± 5.3	c: 8.9 ± 8.09 d: 9.07 ± 8.1	e: 9.8 ± 10.44 f: 9.6 ± 10.11	g: 5.2 ± 4.7 h: 7.5 ± 6.1	0.03	ns	ns	ns
RNS	a: 11.8 ± 10.3 b: 5.2 ± 5.3	c: 9.6 ± 9.6 d: 9.2 ± 9.08	e: 12.52 ± 10.57 f: 10.9 ± 10.1	g: 5 ± 5.6 h: 5.3 ± 5.06	0.0002	ns	ns	ns
Survivin	a: 17.8 ± 4.8 b: 9 ± 6.2	c: 16.3 ± 6.5 d: 12.3 ± 6.5	e: 19.9 ± 3.3 f: 15.1 ± 5.1	g: 10.4 ± 6.3 h: 6.5 ± 5.2	< 0.0001	0.031	0.02	ns
Bcl-2	a: 12.1 ± 4.2 b: 6.4 ± 4.5	c: 11.4 ± 5.4 d: 8.2 ± 4	e: 14.1 ± 3.9 f: 9.5 ± 3.5	g: 17.6 ± 0.5 h: 5.7 ± 3.9	0/0001	0.033	0.008	0.0005
CD44	a: 7.9 ± 5.1 b: 4.03 ± 2.4	c: 8.6 ± 4.7 d: 3.5 ± 2.6	e: 10.7 ± 4.7 f: 3.5 ± 1.6	g: 5.3 ± 2.2 h: 3.5 ± 3.5	0.0003	< 0.0001	< 0.0001	0.004
TWIST	a: 10.2 ± 12.2 b: 6.4 ± 7.2	c: 8.6 ± 10.5 d: 9.2 ± 11.3	e: 9.9 ± 11.9 f: 10.7 ± 12.8	g: 6.5 ± 7.4 h: 6.2 ± 7.01	ns	ns	ns	ns
E-cad	a: 12.9 ± 8.4 b: 13.5 ± 5.6	c: 13.6 ± 7.7 d: 12.5 ± 7.3	e: 13.3 ± 8.6 f: 12.4 ± 8.3	g: 14.1 ± 6.2 h: 12.6 ± 4.7	ns	ns	ns	ns
N-cad	a: 10.7 ± 11.6 b: 5.8 ± 8.4	c: 8.6 ± 10.5 d: 9.5 ± 11.4	e: 10.2 ± 11.1 f: 11.4 ± 12.1	g: 6 ± 8.8 h: 5.6 ± 8.1	0.009	ns	ns	ns
PTPN13	a: 9.7 ± 5.9 b: 12.9 ± 6.6	c: 10.8 ± 6.4 d: 11.03 ± 6.3	e: 10.09 ± 6.22 f: 9.3 ± 5.7	g: 12 ± 6.7 h: 14.5 ± 6.3	0.03	ns	ns	ns
SLUG	a: 2.1 ± 1.8 b: 1.1 ± 2.7	c: 1.8 ± 2.04 d: 1.9 ± 2.3	e: 2.05 ± 1.7 f: 2.6 ± 1.7	g: 1.6 ± 2.4 h: 0.2 ± 3.02	ns	ns	ns	ns

Geometric Mean ± Standard Deviation, *: comparison between group a versus group b, +: comparison between group c versus group d, $: comparison between group e versus group f, ^: $: comparison between group g versus group h, FDR correction for multiple comparisons by Benjamini-Hochberg method

**Table 5:** Comparison of cellular factors levels between the groups positive and negative for HPV

Cellular Factors	PCa group (a) vs control (b)	HPV-positive samples (c) vs HPV-negative samples (d)	HPV-positive PCa (e) vs HPV-negative PCa (f)	HPV-positive control (g) vs HPV-negative control (h)	P^*^	P^+^	P^$^	P^
NF-κB	a:19.4 ± 7.4 b:9.5 ± 7	c: 23.5 ± 5.8 d: 13.2 ± 7.9	e: 24.2 ± 6 f: 17 ± 7	g: 20 ± 3.9 h: 8 ± 6.05	< 0.0001	< 0.0001	0.00091.42	0.004
TNF-α	a:17 ± 7.4 b:11.7 ± 6.7	c: 21.1 ± 6 d: 12.8 ± 6.6	e: 22.4 ± 6.4 f: 14.3 ± 6.4	g: 19.5 ± 3.1 h: 10.6 ± 6.3	0.002	< 0.0001	< 0.0001 1.56	0.03
IL-1	a:13 ± 5.7 b:7.8 ± 5.3	c: 17.2 ± 4.1 d: 9.1 ± 5.2	e: 17.3 ± 4.1 f: 10.9 ± 5.1	g: 16.7 ± 4.2 h: 6.5 ± 4.1	0.0002	< 0.0001	0.0003 1.57	0.002
IL-6	a:14.4 ± 6 b:8.9 ± 6.6	c: 18.9 ± 5 d: 10.2 ± 5.8	e: 18.9 ± 5.2 f: 12.2 ± 5.2	g: 19 ± 3.9 h: 7.5 ± 5.6	0.0004	< 0.0001	< 0.0001 2.51	0.0004
IL-8	a: 12.62 ± 6.2 b: 5.68 ± 5.1	c: 17.3 ± 5.1 d:7.6 ± 5.3	e: 17.7 ± 5.4 f: 10.1 ± 5.09	g: 15.5 ± 3.1 h: 4.2 ± 3.5	< 0.0001	< 0.0001	0.004 1.77	0.006
IL-11	a: 15.6 ± 8.3 b: 7.1 ± 6.2	c: 20.52 ± 6.8 d: 9.8 ± 7.4	e: 21.2 ± 7.2 f: 12.8 ± 7.4	g: 17 ± 5.08 h: 5.7 ± 4.1	< 0.0001	< 0.0001	0.02 1.6	ns
VEGF	a: 38.64 ± 10.8 b: 13.7 ± 6.9	c: 48.5 ± 9.5 d: 25.8 ± 12.5	e: 52.58 ± 3.5 f: 35.8 ± 4.2	g: 29.5 ± 1.2 h: 11.9 ± 3.7	< 0.0001	< 0.0001	0.0009 1.46	0.005
TGF-β	a: 15.2 ± 7.6 b: 7.5 ± 5.1	c: 19.2 ± 5.7 d: 10.1 ± 7	e: 19.9 ± 6.1 f: 12.9 ± 7.2	g: 16 ± 1.6 h: 6.2 ± 4.2	< 0.0001	< 0.0001	0.02 1.54	ns
Rb	a: 9.1 ± 5.7 b: 14.06 ± 6.4	c: 2.4 ± 1.08 d: 13.8 ± 4.6	e: 2.6 ± 1.01 f: 12.3 ± 4.1	g: 1.5 ± 1 h: 15.8 ± 4.6	0.0006	< 0.0001	< 0.0001	0.0003
P53	a: 10.4 ± 6.7 b: 13.9 ± 6.6	c: 3 ± 1.3 d:14.7 ± 5.2	e: 3.2 ± 1.35 f: 14.03 ± 5.2	g: 2 ± 0.8 h: 15.68 ± 5.09	0.04	< 0.0001	< 0.0001	0.002
ROS	a: 10.2 ± 9.7 b: 6.09 ± 5.3	c: 22.3 ± 5.7 d: 3.7 ± 2.7	e: 23.4 ± 5.5 f: 3.1 ± 2.04	g: 17 ± 3.6 h: 4.5 ± 3.3	0.03	< 0.0001	< 0.0001 7.5	ns
RNS	a: 11.8 ± 10.3 b: 5.2 ± 5.3	c: 19.7 ± 8.9 d: 5.9 ± 6.5	e: 20.2 ± 9.7 f: 7.7 ± 7.8	g: 17.2 ± 2.6 h: 3.5 ± 2.8	0.0002	< 0.0001	0.0004 2.6	0.003
Survivin	**a:** 17.8 ± 4.8 **b:** 9 ± 6.2	c: 22 ± 3.2 d: 12.1 ± 5.8	e: 21.9 ± 3.2 f: 15.8 ± 4.1	g: 22.7 ± 3.5 h: 7.03 ± 3.2	< 0.0001	< 0.0001	0.0006	< 0.0001
Bcl-2	**a:** 12.1 ± 4.2 **b:** 6.4 ± 4.5	c: 15.9 ± 3.4 d: 8.1 ± 3.9	e: 15.6 ± 3.7 f: 10.4 ± 3.4	g: 17.3 ± 0.8 h: 4.9 ± 1.9	0/0001	< 0.0001	0.002	< 0.0001
CD44	a: 7.9 ± 5.1 b: 4.03 ± 2.4	c: 7.8 ± 5.1 d: 6.1 ± 4.5	e:7.9 ± 5.4 f: 8 ± 5.01	g: 7.5 ± 3.8 h: 3.5 ± 1.8	0.0003	ns	ns	ns
TWIST	a: 10.2 ± 12.2 b: 6.4 ± 7.2	c: 25.7 ± 7.5 d: 3.1 ± 2.6	e: 26.2 ± 8.1 f: 2.4 ± 1.3	g: 23 ± 1.6 h: 4.03 ± 3.5	ns	< 0.0001	< 0.0001	ns
E-cad	a: 12.9 ± 8.4 b: 13.5 ± 5.6	c: 3.4 ± 2.08 d: 16.5 ± 5.6	e: 3.5 ± 2.2 f:17.5 ± 6.2	g: 3 ± 0.8 h: 15.07 ± 4.2	ns	< 0.0001	< 0.0001	< 0.0001
N-cad	a: 10.7 ± 11.6 b: 5.8 ± 8.4	c: 25.8 ± 7.6 d:3.2 ± 2.4	e: 25.7 ± 8.09 f: 3.4 ± 2.1	g: 26.2 ± 6.2 h: 2.9 ± 2.7	0.009	< 0.0001	< 0.0001	0.004
PTPN13	a: 9.7 ± 5.9 b: 12.9 ± 6.6	c: 4.4 ± 5.6 d: 13.1 ± 5	e: 5 ± 6.01 f:12.08 ± 4.4	g: 2 ± 0.8 h: 14.5 ± 5.5	0.03	< 0.0001	0.0007	0.004
SLUG	a: 2.1 ± 1.8 b: 1.1 ± 2.7	c: 3.8 ± 1.4 d: 1.01 ± 1.9	e: 3.9 ± 1.4 f: 1.2 ± 1.3	g: 3.4 ± 1.2 h: 0.6 ± 2.6	ns	< 0.0001	< 0.0001	Ns

Geometric Mean ± Standard Deviation, *: comparison between group a versus group b, +: comparison between group c versus group d, $: comparison between group e versus group f, ^: $: comparison between group g versus group h, FDR correction for multiple comparisons by Benjamini-Hochberg method

**Table 6:** Comparison of cellular factors levels between the co-infection and mono-infection groups

Cellular Factors	Co-infected PCa (a) vs not co-infected PCa (b)	Co-infected Positive control (c) vs not co-infected control (d)	Co-infected PCa (a) vs HPV-mono-infected PCa (e)	Co-infected PCa (a) vs EBV-mono-infected PCa (f)	Co-infected PCa (a) and non-HPV/non-EBV PCa (g)
NF-κB	a: 26.9 ± 3.1	c: 21.6 ± 2.5	a: 26.9 ± 3.1	a: 26.9 ± 3.1	a: 26.9 ± 3.1
	b: 17.8 ± 7.1	d: 8.2 ± 6	e: 20.3 ± 7.2	f: 18.4 ± 3.2	g: 12 ± 8
	P^*^ (0.0004)	P^+^ (0.004)	P^$^ (ns)	P^ (ns)	P^#^ (0.0001)
TNF-α	a: 25.5 ± 2.7	c: 19.5 ± 3.1	a: 25.5 ± 2.7	a: 25.5 ± 2.7	a: 25.5 ± 2.7
	b: 15.2 ± 6.8	d: 10.6 ± 6.3	e: 19 ± 7.6	f: 16.2 ± 4.8	g: 10.2 ± 6.3
	P^*^ (0.001)	P^+^ (ns)	P^$^ (ns)	P^ (0.04)	P^#^ (0.0001)
IL-1	a: 18.4 ± 3.6	c: 18.3 ± 3.5	a: 18.4 ± 3.6	a: 18.4 ± 3.6	a: 18.4 ± 3.6
	b: 14.2 ± 4.4	d: 6.7 ± 4.2	e: 16.1 ± 4.6	f: 12.5 ± 4.2	g: 7.1 ± 4
	P^*^ (ns)	P^+^ (0.004)	P^$^ (ns)	P^ (ns)	P^#^ (0.0001)
IL-6	a: 21.3 ± 2.1	c: 19 ± 3.9	a: 21.3 ± 2.1	a: 21.3 ± 2.1	a: 21.3 ± 2.1
	b:12.2 ± 5.2	d: 7.5 ± 5.6	e: 16.3 ± 6.5	f: 13.3 ± 5.2	g: 8.2 ± 1.6
	P^*^ (0.004)	P^+^ (0.003)	P^$^ (ns)	P^ (ns)	P^#^ (0.001)
IL-8	a: 17.3 ± 4.9	c: 16.3 ± 3.2	a: 17.3 ± 4.9	a: 17.3 ± 4.9	a: 17.3 ± 4.9
	b: 11.6 ± 6.1	d: 4.5 ± 3.8	e: 18.2 ± 6.1	f: 4.8 ± 3.9	g: 9.1 ± 3.6
	P^*^ (ns)	P^+^ (0.009)	P^$^ (ns)	P^ (0.01)	P^#^ (ns)
IL-11	a: 21.8 ± 6.4	c: 17 ± 5	a: 21.8 ± 6.4	a: 21.8 ± 6.4	a: 21.8 ± 6.4
	b: 14.3 ± 8.1	d: 6.1 ± 5.4	e: 20.6 ± 8.2	f: 14.3 ± 8.1	g: 10.7 ± 5.9
	P^*^ (ns)	P^+^ (ns)	P^$^ (ns)	P^ (ns)	P^#^ (0.04)
IL-17	a: 15.2 ± 1.8	c: 13 ± 2	a: 15.2 ± 1.8	a: 15.2 ± 1.8	a: 15.2 ± 1.8
	b: 8.7 ± 3.3	d: 3.4 ± 2.7	e: 13.2 ± 2.4	f: 8 ± 2.7	g: 7.3 ± 2.4
	P^*^ (0.008)	P^+^ (0.002)	P^$^ (ns)	P^ (0.006)	P^#^ (0.003)
VEGF	a: 53.6 ± 3.8	c: 29.6 ± 1.5	a: 53.6 ± 3.8	a: 53.6 ± 3.8	a: 53.6 ± 3.8
	b: 38.7 ± 7.3	d: 12.5 ± 4.8	e: 51.44 ± 3	f: 35.8 ± 4.7	g: 35.8 ± 3.6
	P^*^ (0.04)	P^+^ (ns)	P^$^ (ns)	P^ (0.02)	P^#^ (0.02)
TGF-β	a: 21.5 ± 4.5	c: 16 ± 2	a: 21.5 ± 4.5	a: 21.5 ± 4.5	a: 21.5 ± 4.5
	b: 13.9 ± 7.5	d: 6.6 ± 4.5	e: 18.2 ± 7.3	f: 13.3 ± 3.5	g: 11 ± 5.6
	P^*^ (ns)	P^+^ (ns)	P^$^ (ns)	P^ (0.03)	P^#^ (0.01)
Rb	a: 2.3 ± 1	c: 1.6 ± 1.1	a: 2.3 ± 1	a: 2.3 ± 1	a: 2.3 ± 1
	b: 10.5 ± 5.3	d: 15.3 ± 5.2	e: 2.7 ± 0.9	f: 12.4 ± 4.7	g: 12.3 ± 3.3
	P^*^ (< 0.0001)	P^+^ (< 0.0001)	P^$^ (ns)	P^ (< 0.0001)	P^#^ (< 0.0001)
P53	a: 2.6 ± 0.9	c: 2.3 ± 0.5	a: 2.6 ± 0.9	a: 2.6 ± 0.9	a: 2.6 ± 0.9
	b: 12.1 ± 6.2	d: 15.1 ± 5.7	e: 3.7 ± 1.4	f: 13.3 ± 5.4	g: 14.9 ± 5
	P^*^ (< 0.0001)	P^+^ (0.0006)	P^$^ (ns)	P^ (< 0.0001)	P^#^ (< 0.0001)
ROS	a: 24.5 ± 5.8	c: 15.6 ± 3	a: 24.5 ± 5.8	a: 24.5 ± 5.8	a: 24.5 ± 5.8
	b: 6.6 ± 8	d: 5.1 ± 4.4	e: 22.2 ± 5.3	f: 3.4 ± 1.9	g: 2.6 ± 2.1
	P^*^ (< 0.0001)	P^+^ (ns)	P^$^ (ns)	P^ (< 0.0001)	P^#^ (< 0.0001)
RNS	a: 20 ± 9.4	c: 17.3 ± 3.2	a: 20 ± 9.4	a: 20 ± 9.4	a: 20 ± 9.4
	b: 10.1 ± 9.7	d: 4 ± 3.7	e: 20.4 ± 10.6	f: 9.2 ± 9.3	g: 5.6 ± 4.3
	P^*^ (0.02)	P^+^ (0.01)	P^$^ (ns)	P^ (0.02)	P^#^ (0.004)
Survivin	a: 22.5 ± 1	c: 21.3 ± 0.5	a: 22.5 ± 1	a: 22.5 ± 1	a: 22.5 ± 1
	b: 13.7 ± 4.9	d: 7.4 ± 4	e: 19.5 ± 1.8	f: 17.2 ± 4.3	g: 12.3 ± 4.7
	P^*^ (< 0.0001)	P^+^ (< 0.0001)	P^$^ (ns)	P^ (ns)	P^#^ (< 0.0001)
Bcl-2	a: 14.1 ± 1.2	c: 15.4 ± 1.3	a: 14.1 ± 1.2	a: 14.1 ± 1.2	a: 14.1 ± 1.2
	b: 8.8 ± 3	d: 5 ± 2.6	e: 13.5 ± 1	f: 8 ± 2.1	g: 7.2 ± 1.9
	P^*^ (0.005)	P^+^ (0.0004)	P^$^ (ns)	P^ (0.005)	P^#^ (0.001)
CD44	a: 10.6 ± 5.4	c: 9.3 ± 1.5	a: 10.6 ± 5.4	a: 10.6 ± 5.4	a: 10.6 ± 5.4
	b: 7.2 ± 4.8	d: 3.5 ± 1.7	e: 3.7 ± 1.4	f: 10.8 ± 4.4	g: 3.9 ± 2
	P^*^ (ns)	P^+^ (ns)	P^$^ (0.03)	P^ (ns)	P^#^ (0.01)
TWIST	a: 26.8 ± 6.7	c: 23 ± 2	a: 26.8 ± 6.7	a: 26.8 ± 6.7	a: 26.8 ± 6.7
	b: 6.8 ± 10.1	d: 4.6 ± 4.9	e: 25.6 ± 9.9	f: 2.5 ± 1.3	g: 2.3 ± 1.4
	P^*^ (< 0.0001)	P^+^ (0.009)	P^$^ (ns)	P^ (< 0.0001)	P^#^ (< 0.0001)
E-cad	a: 2.7 ± 0.6	c: 2.6 ± 0.5	a: 2.7 ± 0.6	a: 2.7 ± 0.6	a: 2.7 ± 0.6
	b: 15.1 ± 7.7	d: 14.6 ± 4.6	e: 4.5 ± 3	f: 18 ± 5.8	g: 16.8 ± 6.9
	P^*^ (< 0.0001)	P^+^ (0.007)	P^$^ (ns)	P^ (< 0.0001)	P^#^ (< 0.0001)
N-cad	a: 25.9 ± 6.8	c: 25.3 ± 7.3	a: 25.9 ± 6.8	a: 25.9 ± 6.8	a: 25.9 ± 6.8
	b: 7.6 ± 9.8	d: 3.8 ± 5.5	e: 25.6 ± 9.7	f: 3.4 ± 1.8	g: 3.4 ± 2.5
	P^*^ (0.001)	P^+^ (0.02)	P^$^ (ns)	P^ (0.0004)	P^#^ (0.0003)
PTPN13	a: 6.2 ± 7.6	c:2 ± 1	a: 6.2 ± 7.6	a: 6.2 ± 7.6	a: 6.2 ± 7.6
	b: 10.5 ± 5.3	d: 14.1 ± 5.8	e: 3.6 ± 3.6	f: 12.7 ± 4.7	g: 12.5 ± 4
	P^*^ (ns)	P^+^ (0.002)	P^$^ (ns)	P^ (0.03)	P^#^ (0.03)
SLUG	a: 3.5 ± 1.6	c: 3.2 ± 1.4	a: 3.5 ± 1.6	a: 3.5 ± 1.6	a: 3.5 ± 1.6
	b: 1.8 ± 1.8	d: 0.96 ± 2.8	e: 4.3 ± 1.1	f: 0.9 ± 1.4	g: 1 ± 1.1
	P^*^ (ns)	P^+^ (ns)	P^$^ (ns)	P^ (0.004)	P^#^ (0.01)

Geometric Mean ± Standard Deviation, *: comparison between group a versus group b, +: comparison between group c versus group d, $: comparison between group e versus group f, ^: $: comparison between group g versus group h, FDR correction for multiple comparisons by Benjamini-Hochberg method

**Table 7:** Spearman’s correlation coefficient between the cellular factors with the HPV and EBV genes

	LMP-1	LMP-2	EBER 1	EBER 2	E2	E6	E7
NF-κB	0.6^****^	0.43^***^	0.33^**^	0.31^**^	0.37^**^	0.64^****^	0.64^****^
TNF-α	0.49^**^	0.18 ^ns^	0.19 ^ns^	0.28^*^	0.271^**^	0.50^***^	0.504^***^
IL-1	0.501^**^	0.42^**^	0.34^**^	0.22^*^	0.40^***^	0.533^***^	0.529^***^
IL-6	0.66^***^	0.41^**^	0.11 ^ns^	0.28^*^	0.309^**^	0.444^***^	0.435^***^
IL-8	0.54^***^	0.11 ^ns^	0.07^ns^	0.08 ^ns^	0.393^**^	0.603^***^	0.596^***^
IL-11	0.55^***^	0.3^*^	0.34^*^	0.136 ^ns^	0.341^***^	0.504^***^	0.509^***^
IL-17	0.56^***^	0.41^**^	0.104 ^ns^	0.09 ^ns^	0.420^***^	0.726^***^	0.724^***^
VEGF	0.57^****^	0.39^*^	0.302^*^	0.07 ^ns^	0.323^**^	0.595^***^	0.604^***^
TGF-β	0.51^***^	0.25^*^	0.103 ^ns^	−0.08 ^ns^	0.31^**^	0.55^***^	0.59^****^
Rb	−0.47^***^	0.39^**^	0.28^**^	0.22^*^	−0.553^***^	−0.751^***^	−0.751^***^
P53	−0.51^***^	−0.401^*^	−0.201^*^	−0.05 ^ns^	−0.456^***^	−0.712^***^	−0.715^***^
Survivin	0.76^****^	0.39^**^	0.308^**^	0.17 ^ns^	0.528^***^	0.757^***^	0.761^***^
Bcl-2	0.49^***^	0.39^**^	0.102 ^ns^	−0.03 ^ns^	0.556^***^	0.743^***^	0.743^***^
ROS	0.46^**^	0.29^*^	0.09 ^ns^	0.105 ^ns^	0.525^***^	0.763^***^	0.763^***^
RNS	0.301^*^	0.08 ^ns^	0.02 ^ns^	0.106 ^ns^	0.460^***^	0.654^***^	0.652^***^
CD44	0.8^****^	0.68^****^	0.48^***^	0.58^****^	0.21^*^	0.34^**^	0.29^**^
TWIST	0.53^****^	0.44^***^	0.44^***^	0.045 ^ns^	0/53^**^	0/83^****^	0/86^****^
E-cad	−0.5^***^	−0.2^*^	− 0.11^*^	− 0.096 ^ns^	− 0/47^**^	− 0/71^***^	−0/71^***^
N-cad	0.51^****^	0.41^**^	0.42^**^	0.206 ^*^	0/51^**^	0/85^****^	0/87^****^
PTPN13	−0.409^***^	− 0.15 ^ns^	−0.15 ^ns^	− 0.01 ^ns^	− 0/48^**^	−0/41^*^	− 0/38^*^
SLUG	0.35^*^	0.05 ^ns^	0.05 ^ns^	0.10 ^ns^	0/35^*^	0/46^**^	0/46^**^

ns: not significant, * p < 0.05; ** *p* < 0.01; *** p < 0.001, **** < 0.0001

Compared with the EBV-negative group, EBV-negative PCa group and EBV-negative control group, the expression level of VEGF, IL-6, IL-1, NF-κB and TNF-α was higher in EBV-positive control, EBV-positive PCa and EBV-positive groups, respectively (Table 4). As shown in Table 7, there was a strongest correlation between LMP-1 and IL-6 (R = 0.66, *P* = 0.001). Table 7 presents more details***.*** Since some of the samples studied were both infected with the EBV and infected with the HPV (HPV/EBV coinfection), the question may now be whether the increased expression of inflammatory factors was the result of the effect of EBV-infection or the presence of HPV-infection and/or effect of HPV/EBV-co-infection? Therefore, as can be seen in Table 6, the level of inflammatory factors was compared between the co-infected and mono-infected samples. Some inflammatory factors (VEGF, IL-17, IL-6, TNF-α and NF-κB) had statistically higher expression level in the HPV/EBV co-infected PCa group when comparing with not co-infected PCa group. It is worth mentioning, not-coinfected PCa group including mono HPV-infected PCa samples, mono EBV-infected PCa samples, and non HPV and non EBV PCa samples. The results showed that all inflammatory factors had an increase in the mean expression level in the HPV/EBV co-infected PCa group when comparing with the mono HPV infected PCa group, but this increase was not significant. Although increase in mean expression was not statistically significant, can it be concluded that cause is due to a presence of EBV infection? In comparing inflammetory factors between HPV/EBV co-infection PCa group with mono EBV infected PCa group, it was observed that there was a significant higher mean expression level of TNF-α, IL-17, IL-8, RNS, ROS, TGF-β and VEGF in coinfected group than in the mono EBV infected group, but the two groups showed no significant difference in the mean expression level of IL-11, IL-6, IL-1 and NF-κB (*P* > 0.05). Therefore, it can be concluded that an elevated expression level of RNS, ROS, TGF-β, VEGF, TNF-α, IL-17 and IL-8 probably more due to the presence of HPV infection than to EBV infection and also an elevated expression level of IL-11, IL-6, IL-1 and NF-κB factors may be due to the simultaneous presence of HPV and EBV infections. In addition, the level of all inflammatory factors except IL-8 in HPV/EBV co-inffected PCa group was significantly higher than in non-HPV and non-EBV PCa group (Table 6).

### Comparison of the expression pattern of apoptosis-related, tumor suppressor, Anoikis-related factors and CD44 among the study groups

Following the identification of differences in the expression levels of inflammatory-related mediators between studies groups, we examined the association between the concentrations of antiapoptotic factors (survivin and Bcl-2), tumor suppressor factors (p53 and Rb), anoikis-related mediators (SLUG, PTPN13, N-cadherin, E-cadherin and TWIST) and CD44 (as a cell adhesion glycoprotein) in the samples infected with HPV, the samples infected with EBV, the samples coinfected with HPV/EBV, and control groups. In comparison with the control group, the p53 and Rb expression levels were significantly lower and also Bcl-2, Survivin and CD44 levels were higher significantly in the PCa group. Furthermore, among anoikis-related factors, only N-cad and PTPN13 levels significantly were higher and lower, respectively, in the PCa group when comparing with the control group (Table 4). Analysis of the results also showed no significant difference in the expression level of tumor suppressor proteins and anoikis-related factors between EBV-positive samples and EBV-negative samples. However, the concentrations of CD44, Survivin and Bcl-2 in the EBV-positive samples were significantly higher compared to the EBV-negative samples (Table 4). As well, a significant positive correlation was observed between the EBV-LMP1 gene expression levels and the expression levels of CD44 (r = 0.8, *P* < 0.0001) and Survivin (r = 0.76, P < 0.0001) (Table 7). As seen in Table 5, there was a significant reduction and elevation in the concentration of tumor suppressor proteins (p53 and Rb) and the anti-apoptotic proteins (survivin and Bcl-2), respectively, in the HPV-positive groups in comparison with the HPV-negative groups. Also, the concentration of E-cad and PTPN13 proteins was significantly lower and the concentration of N-cad, SLUG, TWIST was significantly higher in the HPV-positive groups when comparing with the HPV-negative groups. In addition, no statistically significant difference was found in the expression pattern of CD44 between HPV-positive groups when comparing with the HPV-negative groups (*P* > 0.05). As shown in Table 7, there was a significantly positive correlation between the E7 and E6 level and Survivin (r:0.75, and 0.76, respectively, *P* < 0.001), Bcl-2 (r:0.74, and 0.74, respectively, P < 0.001), TWIST (r:0.83, and 0.86, respectively, P < 0.0001) and N-cad (r:0.85, and 0.87, respectively, P < 0.0001). Besides, there was a significantly negative correlation between E7 and E6 level and Rb (r:-0.75, and − 0.75, respectively, P < 0.0001), p53 (r:-0.71, and − 0.71, respectively, P < 0.0001), E-cad (r:-0.71, and − 0.71, respectively, P < 0.0001).

Based on correlation results in Table 6, significantly lower and higher expression levels were reported for the tumor suppressor proteins (p53 and Rb) and Bcl-2 in the HPV/EBV co-infection PCa group, respectively in compared to the mono EBV positive PCa group, and the non-HPV/non-EBV PCa group. As well, the mean expression level of these factors between HPV/EBV co-infected PCa and mono HPV-infected PCa groups are different which it probably due to the presence of the EBV infection and it can contribute to the progression of prostate cancer by modulation of cellular factors. In comparing Survivin between HPV/EBV co-infection PCa group with mono HPV- and mono-EBV-infected PCa group, it was observed that no significant difference between these groups (P > 0.05). Therefore, it can be concluded which high concentration of Survivin in coinfected PCa group maybe can due to the simultaneous presence of HPV and EBV infections.

Expression level of CD44 in PCa and EBV-positive groups were significantly higher than control and EBV-negative groups, respectively (Table 4). As shown in Table 6, the mean CD44 expression level was significantly different only between the two groups (HPV/EBV coinfection PCa group versus mono HPV-positive PCa group), but not between coinfection PCa group and mono EBV-positive PCa group. Thus, the increase in mean expression level of CD44 in the coinfection group is most likely due to the presence of EBV virus.

According to the results of Table 4, among anoikis-related factors, the expression level of N-cad and PTPN13 in the PCa group is significantly different from the control. Also, no significant difference was seen in the mean expression level of anoikis-related factors between EBV-positive and EBV-negative samples (Table 4). Conversely, a significant difference was reported between the mean expression levels of all of these factors in HPV-positive compared to HPV-negative samples (Table 5). The mean expression level of TWIST, N-cad and SLUG in HPV/EBV co-infected PCa group was higher compared to EBV/HPV negative PCa, mono EBV-infected PCa, non HPV/non EBV PCa groups. As shown in Table 7, a significant negative correlation was seen between the expression level of E-cad with E7 and E6 (r = − 0/71, *P* < 0.0001), and also between E7 and E6 levels with the TWIST (r = 0/83, r = 0/86, respectively, P < 0/0001) and with the N-cad (r = 0/85, r = 0/87, respectively, P < 0/0001). More information is given in Table 7.

In compared HPV/EBV coinfected PCa group with coinfection negative PCa, mono EBV-positive PCa and non-HPV/non-EBV PCa groups, the E-cad level and the TWIST and N-cad levels had significantly decreased and increased expression, respectively (Table 6). However, no significant difference was found in the mean expression level of Anoikis-related factors between the mono HPV positive PCa group and the co-infection positive PCa group. In conclusion, the HPV infection is probably the cause of these changes and HPV plays important role in contribute to anokis resistance than the EBV virus.

## Discussion

The present study aimed to find differences among EBV-positive samples, HPV-positive samples, HPV/EBV-coinfected samples, and control samples concerning the expression levels of EBV and HPV genes, HPV integration physical status, and expression levels of some inflammatory, tumor-suppressor, antiapoptotic, and anoikis-related mediators, including IL-17, IL-11, IL-6, IL-1, IL-8, TNF-α, NF-κB, VEGF, TGF-β, ROS, RNS, Rb, p53, survivin, Bcl-2, CD44, Twist, E-cadherin, N-cadherin, PTPN13, and Slug.

Evidence shows that viral infections account for about 12% of all cancers globally [11, 12]. HPV and EBV account for 38% of all virus-related cancers [43]. Besides, the co-presence of HPV and EBV has been reported in some human malignancies, such as cervical cancer, breast cancer, nasopharyngeal carcinoma (NPC), and PCa [18]. In the present study, HPV and EBV were isolated from 31.3 and 49.3% of patients in the PCa group, as well as 15 and 40% of subjects in the control group, respectively. The EBV/HPV coinfection was found in 14.9% of PCa patients and 7.5% of control subjects. Based on the results, the high-risk strains of HPV, that is, HPV 16 and HPV 18, were responsible for 50 and 30% of HPV/EBV-coinfected PCa cases, respectively.

So far, the EBV infection has been reported in prostate tissues. In a previous study, the presence of EBV was reported in 8.8% (31 out of 352) of malignant and benign prostate tissue samples in Sweden [27], and in 8% of tissues (16 out of 200) in the United States [28]. Another study reported EBV infection in almost 37% of PCa patients (*n* = 19) [26]. Besides, high levels of HPV-18 and EBV (EBNA1) gene sequences were detected in a previous study, and these sequences were almost equally found in normal and benign PCa samples [29]. The EBV/HPV coinfection was significantly more prevalent in PCa patients (55%) as compared to benign PCa (15%) and normal prostate (30%) cases [29]. It seems that EBV and HPV act simultaneously increasing the cultured cervical cell proliferation and the same behaviors may also occur in prostate epithelial cells [29, 44], which is in line with the present experiments on high-risk HPVs and EBV in PCa patients.

Some studies have shown that EBV infection in the cervix accelerates the integration of HPV genomes into the cervical cell genomes [21, 45, 46]. Consistent with this hypothesis, our results showed maximum HPV genome integration in the HPV/EBV-coinfected PCa group (8/10; 80%). Also, the rate of purely integrated HPV (47.4%) was significantly higher than purely episomal HPV (5.2%) in tumor tissues positive for HPV. Moreover, the frequency of purely integrated HPV was significantly higher in HPV/EBV-coinfected PCa samples, compared to mono HPV-infected PCa samples (*P* = 0.0009).

Since no study has yet investigated the effect of HPV/EBV coinfection on PCa development, we compared our findings with the results reported for other cancers. The EBV probably acts as a co-factor for HPV to induce the uterine cervix pathology, as shown by Szkaradkiewicz et al. [47] who reported a possible sexual transmission route for EBV. It has been also found that sexually transmissible infections are associated with the increased risk of PCa [48]. Additionally, a recent meta-analysis by de Lima et al. showed that EBV infection was associated with a two-fold increase in the risk of precancerous cervical lesions and a four-fold increase in the risk of cervical cancer in HPV-positive women [45]. However, in the current study, the presence of EBV, HPV, and HPV/EBV coinfection had no significant associations with PCa (*P* = 0.35, *P* = 0.06, and *P* = 0.34, respectively).

A previous study showed that EBV LMP1, in combination with HPV16 E6 proteins in transformed mouse embryonic fibroblasts, caused a decrease in the residues of DNA damage response, including p27, pRb, and p53, and led to an increase in the level of checkpoint kinase 1 (Chk1) and Akt, MAPK, and NF-κB signaling [49]. Similarly, in the present study (as described in the results section 3.5), the mean expression levels of p53 and Rb in the coinfected group were lower than the mono HPV- and mono EBV-infected groups. There was no significant difference between the coinfected group and the mono HPV-infected group, while there was a significant difference between the coinfected group and the mono EBV-infected group.

In the current study, there was no significant difference between EBV-positive and EBV-negative samples. However, a significant inverse correlation was found between the expression levels of HPV E7 and E6 mRNA and the expression levels of p53 and Rb (Table 7). Therefore, it can be concluded that the lower concentrations of Rb and p53 in the coinfected PCa group were probably due to the co-presence of HPV and EBV infections; nonetheless, the effect of HPV infection might be more significant than EBV infection.

The main pathogenic mechanism in the development of cancers, caused by EBV and HPV, is the induction of a cytokine effect and chronic inflammation [15] nevertheless, the role of HPV/EBV coinfection in PCa is unknown. As mentioned in the results section (*section 3.4*; Tables 6 and 7), there was a possible association between inflammation and the co-presence of HPV and EBV in PCa cases. Also, inflammatory factors showed higher mean expression levels in the HPV/EBV-coinfected group as compared to the mono HPV- and mono EBV-infected groups; however, no statically significant difference was found between the coinfected and mono HPV-infected groups. Based on the comparison of the mean expression of inflammatory factors between the groups with coinfection and mono-infection (*section 3.4;* Table 6), it can be concluded that HPV/EBV coinfection increases the mean expression of inflammatory factors, compared to mono-HPV and mono-EBV infections.

It has been hypothesized that the co-presence of two or more infectious agents may lead to the increased levels of systemic inflammatory cytokines [50–52] Also, some factors, such as lifestyle (e.g., smoking and alcohol consumption), genetic factors, and coinfection with microbial agents, can significantly increase the possibility of developing persistent infection with high-risk HPV types [53–56]. Therefore, coinfection with HPV and EBV and integration of HPV genome may explain the mechanism of HPV persistence in inflammatory conditions; however, further experimental investigation is needed in future studies.

In another study by Grace et al., a significant positive correlation was observed between HPV-induced squamous cell carcinoma (SCC) and the expression levels of Bcl2/p53 proteins [57] suggesting that the high-risk HPV-E6 oncoprotein could enhance the Bcl-2 protein expression through the blocking of the inhibitory activity of p53 on Bcl-2 [58]. Survivin and Bcl-2 (as antiapoptotic proteins) are critical factors in regulating the progression of cell cycle and preventing apoptosis [59]. In a study by Guo et al., the upregulation of p53-induced survivin was promoted by LMP1 through the increased activity of survivin promoters and increased p53-survivin DNA binding; therefore, the complexity of p53 regulation in survivin is associated with viral LMP1 oncoproteins in NPC. Overall, their model of p53-induced G1/S cell cycle progression could upregulate the LMP1-mediated expression of survivin in the pathogenesis of NPC [60]. Moreover, the LMP1-induced upregulation of Bcl-2 has been reported in B cells [61]. In a study of Muzio et al., evaluation of survivin expression, oral premalignant lesions, and oral carcinoma in the presence of HPV infection showed significantly higher expression levels of survivin in HPV-positive samples, compared to the HPV-negative ones. Evidence suggests that the expression of survivin may be directly or indirectly influenced by HPV [62]. In the present study, it was observed that the expression levels of survivin and Bcl-2 were significantly higher in the HPV-infected, EBV-infected, and HPV/EBV-coinfected groups, compared to HPV-negative, EBV-negative, and non-coinfected groups, respectively. Additionally, there was a direct association between the expression levels of E6 and E7 and the expression levels of survivin and Bcl-2; also, a direct association was found between the expression levels of LMP-1 and survivin. It seems that the EBV/HPV coinfection may initiate the neoplastic transformation of carcinogenesis [25]. Also, an in-vivo interaction occurs between HPV and EBV and between EBV and HPV oncoproteins [44]. These results suggest that the co-presence of HPV and EBV infections may lead to the resistance of cancer prostate cells to apoptosis, although the effect of HPV infection might be greater than EBV infection. However, to prove these, more experimental research is needed, including wet-laboratory work.

In a study by Castilla et al., PTPN13 gene silencing elevated the expression of invasion-related genes in PCa cells [63]. Also, HPV-E6 triggers anchorage-independent growth in human epithelial cells through PTPN13 loss [64, 65]. The present study analyzed the expression levels of Slug, Twist, N-cadherin, E-cadherin, and PTPN13 proteins in prostate tissue lysates. As mentioned before, the mean expression levels of N-cadherin, Slug, and Twist were significantly higher in HPV-positive cases, compared to the HPV-negative group, while PTPN13 and E-cadherin were significantly downregulated in the HPV-positive PCa group, compared to the HPV-negative PCa group (Table 5). Based on the present findings, the expression levels of E6 and E7 had significant negative and positive correlations with E-cadherin and N-cadherin/Twist/Slug, respectively (Table 7). However, the mean CD44 expression was significantly higher in EBV-positive cases, compared to the EBV-negative ones, and there was a positive correlation between the expression levels of LMP-1 and LPM-2 and the expression level of CD44 (Tables 4 and 7).

It has been reported that LMP1 downregulates the E-cadherin expression and upregulates Twist and other transcription factors associated with cell motility [66, 67]. An EBV-protein, i.e., LMP-1, triggers anoikis resistance by inducing the expression of anti-apoptosis proteins, survivin, CD44, inhibitor of DNA binding 1 (ID1), BIM, and ROS [68, 69]. Moreover, the co-expression of E6 and LMP1, compared to the expression of E6 and EBNA1 alone, triggers some processes, including tumor formation, anchorage-independent growth, and resistance to apoptosis and cell proliferation in nude mice [70]. The co-expression of LMP1 and HR-HPV E6 is associated with more aggressive malignant tumors, including cervical SCC and breast adenocarcinoma [71, 72]. In the current study, the levels of Twist and N-cadherin increased in the HPV/EBV-coinfected PCa group, compared to the non-coinfected PCa group, while the expression level of E-cadherin significantly decreased. However, no significant difference was reported in the mean expression of anoikis-related factors between the PCa group positive for coinfection and the mono HPV-infected PCa group. Besides, the mean level of CD44 expression was not significantly different between the HPV/EBV-coinfected PCa group and the mono EBV-infected PCa group. Therefore, the increased expression of CD44 in the coinfected group might be due to the presence of EBV. Based on the findings, co-presence HPV/EBV infection or mono-infection is probably can contribute to these changes (i.e., E-cadherin, N-cadherin, Twist, PTPN13, Slug, CD44, Bcl-2, and Survivin), but further experimental studies are needed to prove the role of viral infections in the regulation of Anoikis molecular pathways and metastasis in PCa [73–78].

The carcinogenic mechanisms of the HPV and the EBV viruses can be different [79–81], for example, the integration of the HPV genome is a critical stage in HPV carcinogenicity [82] while this phenomenon is not observed in EBV. Accumulating evidence suggests that both viruses contribute to tumor progression by affecting the common pathways. Recently, it has been suggested that both HPV and EBV oncoproteins induce the initiation of EMT and the progression of human carcinomas through interactions with the JAK/STAT/SRC, β-catenin, PI3k/Akt/mTOR, and/or RAS/MEK/ERK signaling pathways [83]. The co-presence of HPV and EBV oncoproteins may contribute to cancer progression through EMT initiation [83]. According to the results of the present study, the HPV/EBV coinfection, compared to mono-infections, might lead to more changes in the mean expression of factors involved in inflammation and progression of malignancies, although some changes were not statistically significant. It can be concluded that the HPV/EBV coinfection is likely to affect the pathways involved in tumorigenesis more than mono-HPV and mono-EBV infections. However, further research is needed to confirm this hypothesis.

One of the limitations of this study is that we had to sample a peripheral area of surgically dissected benign prostatic hyperplasia due to lack of access to normal and healthy prostate samples.

## Conclusion

**Fig. 2:**
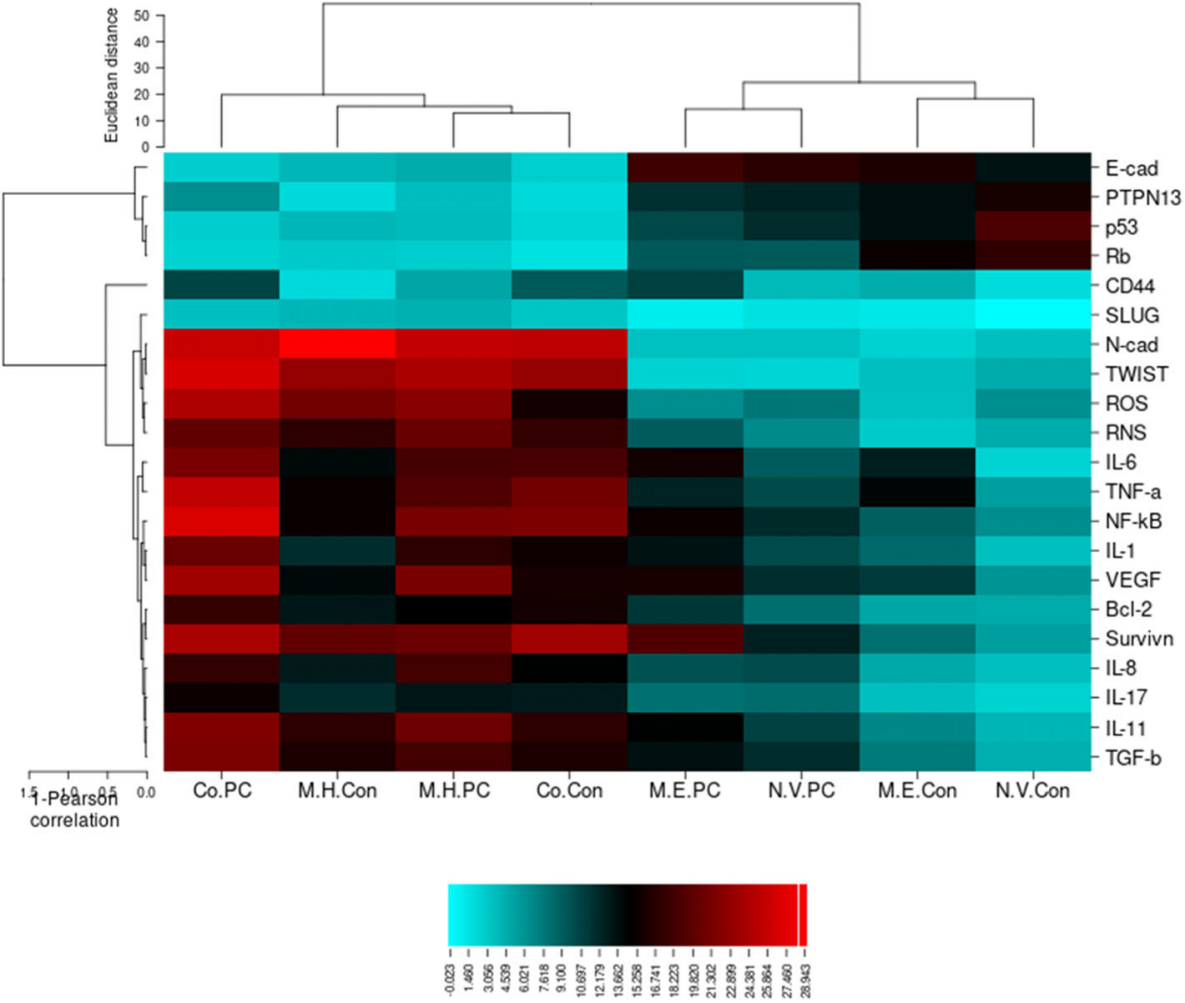
Hierarchical clustering of the differentially expressed Proteins and mRNAs between the studied groups. (M.H.Con: mono HPV-infected control samples, Co.PC: HPV/EBV-Coinfected PCa, M.H.PC: mono HPV-infected PCa samples, Co.Con: HPV/EBV coinfected control samples, M.E.PCa: mono EBV-infected PCa samples, N.V.Con: non-HPV/non-EBV control samples, N.V.PC: non-HPV/non-EBV PCa samples, and M.E.Con: mono EBV-infected control samples). The mean expression level of cellular factors in different groups, indicates that the expression pattern of cellular factors in Co-infected groups and mono-HPV samples is similar

According to the results of the present study, the HPV/EBV coinfection was present in 14.9% of PCa cases, and the high-risk strains (HPV 16 and HPV 18) were responsible for 50 and 30% of PCa samples coinfected with HPV/EBV (*n* = 10), respectively. The maximum percentage of HPV genome integration was found in the HPV/EBV-coinfected PCa group (8/10; 80%). Although there was no statistically significant association between the HPV/EBV co-infection and PCa, the expression profile of some cellular factors involved in inflammation, tumor progression, and metastasis were different between the HPV/EBV-coinfected PCa group with both mono-EBV infection and mono-HPV infection. These differences suggest that the co-presence of these viruses alters the expression patterns of cellular factors, compared to mono-infections (Fig. 2), suggesting the HPV/EBV coinfection as a contributing factor for the development of PCa and indicating the role of EBV in the HPV genome integration. Finally, there are some limitations that were associated with our work. For example, we are not able to perform wet-lab experiments. Hence, we suggest that these experiments be performed in the future researches.

## Supplementary Information

**Additional file 1: Fig. S1.** PCR amplification of the RBV EBER-2 gene. Fig. S2. PCR amplification of EBV genotypes. Fig. S3. Nitrocellulose Strips After Staining.

## Abbreviations

PCa: Prostate Cancer
HPV: Human papilloma virus
EBV: Epstein-Barr Virus
EBER-1: Epstein-Barr Virus-Encoded RNA-1
EBER-2: Epstein-Barr Virus-Encoded RNA-2
LMP-1: Latent Membrane Proteins-1
LMP-2: Latent Membrane Proteins-2
OR: Odds ratio
qRT-PCR: quantitative RT-PCR
RT-PCR: Real-time PCR
Bcl-2: B-cell lymphoma 2
ECM: Extracellular matrix
IL: Interleukin
ELISA: Enzyme-linked immunosorbent assay
NF-κB: Nuclear factor-kB
ROS: Reactive oxygen species
TNF-α: Tumor necrosis factor α
TGF-β: Transforming growth factor β
VEGF: Vascular endothelial growth factor
RB: Retinoblastoma
PTPN13: Protein tyrosine phosphatase non-receptor type 13
