# Supplementary material for: Possible role of HPV/EBV coinfection in anoikis resistance and development in prostate cancer
Source: BMC Cancer. 2021 Aug 17;21:926. doi: 10.1186/s12885-021-08658-y (PMC8369687; doi:10.1186/s12885-021-08658-y)
Supplement: Supplementary file 1 — Additional file 1: Fig. S1. PCR amplification of the RBV EBER-2 gene. Fig. S2. PCR amplification of EBV genotypes. Fig. S3. Nitrocellulose Strips After Staining. [file 12885_2021_8658_MOESM1_ESM.docx]

**Figure 1. PCR amplification of the RBV EBER-2 gene
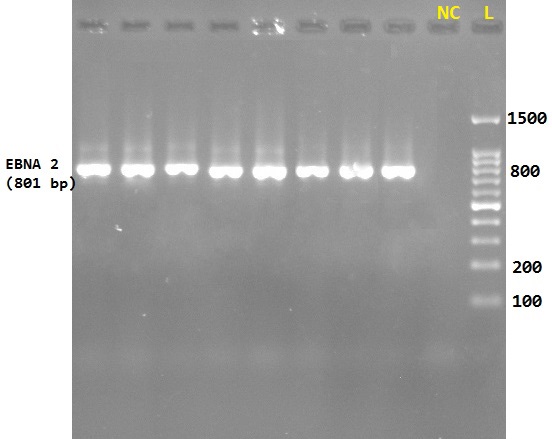
.** The first round of the PCR was amplified by using a specific primer (EBNA-2F: GGAAACCCGTCACTCTC and EBNA-2R: TAATGGCATAGGTGGAATG) which gives an amplicon size of 801 bp. L; ladder (100-1500), NC (negative control).


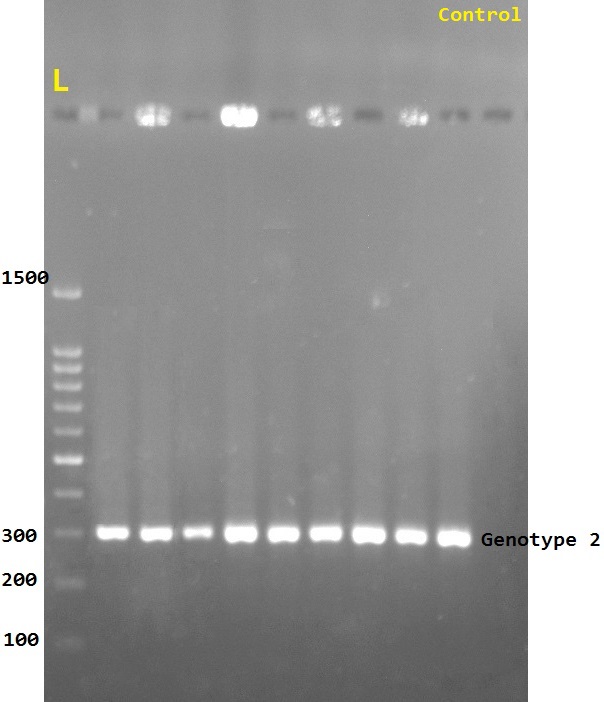
 **Figure 2. PCR amplification of EBV genotypes.** The second round of the RT-PCR was amplified by using forward primer EBNA-2C (AGGGATGCCTGGACACAAGA), which is common to both EBV genotypes 1 and 2, as well as reverse primers EBNA-2G (GCCTCGGTTGTGACAGAG, amplicon size: 250 bp,) and EBNA-2B (TTGAAGAGTATGTCCTAAGG, amplicon size: 300 bp, EBV genotype 2), that are specific primers for genotypes 1 and 2, respectively.


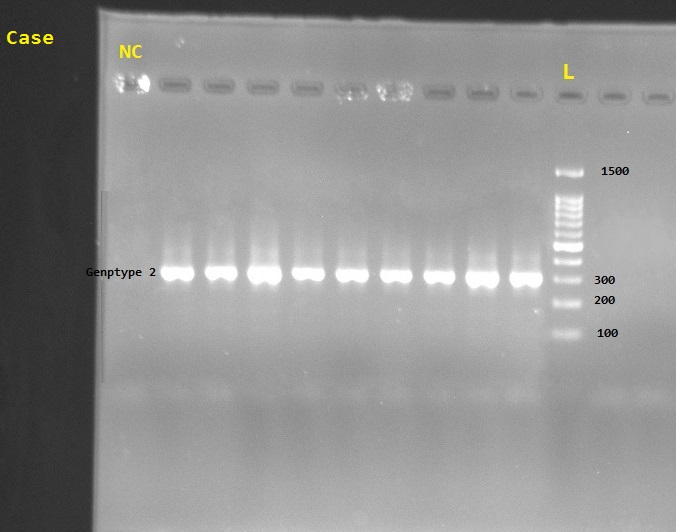
lml


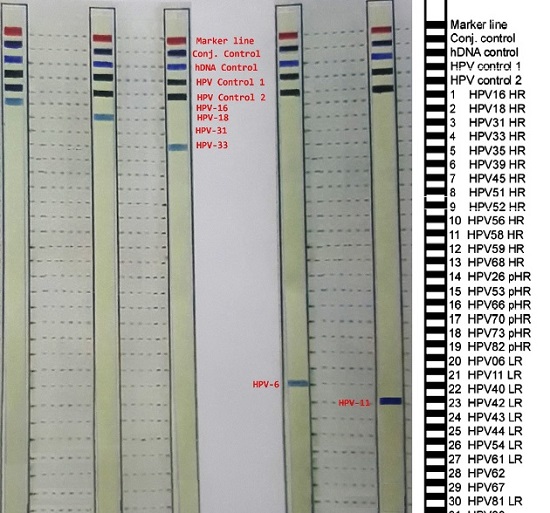


**Figure 3. Nitrocellulose Strips After Staining.** The detected genotypes were HR-HPV types, 16, 18, and 33, as well as LR-HPV 6 and 11.
